# Rarely selected distractors in high stakes medical multiple-choice examinations and their recognition by item authors: a simulation and survey

**DOI:** 10.1186/1472-6920-10-85

**Published:** 2010-11-24

**Authors:** Anja Rogausch, Rainer Hofer, René Krebs

**Affiliations:** 1 Assessment and Evaluation Unit, Institute of Medical Education, Faculty of Medicine, University of Bern, Konsumstrasse 13, CH-3010 Bern, Switzerland

## Abstract

**Background:**

Many medical exams use 5 options for multiple choice questions (MCQs), although the literature suggests that 3 options are optimal. Previous studies on this topic have often been based on non-medical examinations, so we sought to analyse rarely selected, 'non-functional' distractors (NF-D) in high stakes medical examinations, and their detection by item authors as well as psychometric changes resulting from a reduction in the number of options.

**Methods:**

Based on Swiss Federal MCQ examinations from 2005-2007, the frequency of NF-D (selected by <1% or <5% of the candidates) was calculated. Distractors that were chosen the least or second least were identified and candidates who chose them were allocated to the remaining options using two extreme assumptions about their hypothetical behaviour: In case rarely selected distractors were eliminated, candidates could randomly choose another option - or purposively choose the correct answer, from which they had originally been distracted. In a second step, 37 experts were asked to mark the least plausible options. The consequences of a reduction from 4 to 3 or 2 distractors - based on item statistics or on the experts' ratings - with respect to difficulty, discrimination and reliability were modelled.

**Results:**

About 70% of the 5-option-items had at least 1 NF-D selected by <1% of the candidates (97% for NF-Ds selected by <5%). Only a reduction to 2 distractors and assuming that candidates would switch to the correct answer in the absence of a 'non-functional' distractor led to relevant differences in reliability and difficulty (and to a lesser degree discrimination). The experts' ratings resulted in slightly greater changes compared to the statistical approach.

**Conclusions:**

Based on item statistics and/or an expert panel's recommendation, the choice of a varying number of 3-4 (or partly 2) plausible distractors could be performed without marked deteriorations in psychometric characteristics.

## Background

Item writing guidelines for multiple choice questions (MCQs) advise authors to create as many options as feasible [1-3]. Nevertheless it was assumed that, in most cases, 3 plausible options (1 correct option and 2 distractors) may represent a natural limit [4]. Plausible distractors should attract candidates with insufficient knowledge; so non-functional distractors (NF-D) can be defined as being so implausible that only a few candidates (e.g. less than 5%) would select them [4,5].

Various studies have been devoted to 'the optimal number of options' in MCQs, as limiting the number of options could facilitate item writing and reduce the candidates' time to complete the examination. More questions could then be included in an examination to enhance reliability and thereby use testing time more efficiently [e.g. [6,7]]. It could also reduce the influence of testwiseness, as candidates might use cues from the distractors to their advantage [8]. On the other hand, the likelihood that the correct answer may be selected by chance might increase if the number of options is reduced.

Rodriguez summarised existing theoretical and empirical studies that reached the conclusion that 3 options 'are optimal for MC items in most settings' [9]. This meta-analysis, several reviews and studies [e.g. [2,7,10-12]] have all indicated that while mean percentage of correct answers (P-value) may be slightly enhanced due to the reduction in the number of distractors (making the examination easier), mean discrimination and reliability seem to be less affected.

However, omitting NF-Ds requires that item authors can prospectively identify and selectively avoid them. Alternatively, item-writing committees consisting of several experts could review the questions and decide which option to discard. Swanson et al. (2008) concluded from their study that these committees would be sufficiently effective in selecting distractors even without access to response statistics [6].

The number of plausible distractors may vary depending on the educational setting and type of examination [9]. Though the literature suggests that 3 options are optimal, only a few of these analyses have been based on medical examinations [e.g. [6,13,14]]. This might be the reason why many medical exams still use 5 options [e.g. [15]]. Swanson et al. (2008) recommended further studies to determine whether their results [mainly derived from the assessment of skills in diagnostic reasoning (USMLE Step 2 CK)] would be generalisable to other medical topics [6]. Therefore we wanted to investigate which psychometric changes are to be expected if less than 5 options would be used in high stakes medical examinations encompassing several different topics. Additionally, we were interested to determine to what extent authors can correctly detect these NF-Ds and thereby exclude or avoid them during examination preparation in the future.

## Methods

### Database of examinations

The analyses regarding the frequency of NF-Ds were based on the Swiss federal graduation MCQ examinations of 2005, 2006 and 2007. Five annual examinations - each with 120 questions of different item types - cover 9 specialities. The five examinations were conducted and evaluated separately, but the grades could be compensated across the 5 MCQ examinations and 9 oral/practical examinations. In each of the years 2005-2007 more than 600 candidates from all 5 Swiss medical faculties took these exams. We focused our analyses on positively formulated best answer items (type A+). Some of the items were used in more than 1 year; in these cases the first usage of the item was taken into account. Other items had been eliminated after the examination due to identified flaws (i.e. did not influence the candidates' test results); these items were also excluded from our analysis. All data were completely anonymised, aggregated and non-individual-related, so informed consent and ethical approval was dispensable. Analyses regarding possible consequences from a reduction to 4 (or 3) options, respectively, were based on the 5 exams from the year 2005. Our database is presented in table 1.

**Table 1:** Number of candidates and items per discipline included in the analysis

Subject	Number of	2005	2006	2007	Total
Internal medicine/pharmacotherapy	Items	55	50	45	150
	
	Candidates	617	627	644	

Surgery	Items	54	49	63	166
	
	Candidates	618	624	649	

Gynaecology and paediatrics	Items	43	36	44	123
	
	Candidates	615	629	646	

Dermatology, ophthalmology, otorhinolaryngology	Items	57	64	59	180
	
	Candidates	611	632	654	

Social and preventive Medicine	Items	41	38	39	118
	
	Candidates	606	645	648	
***Total***	*Items*	*250*	*237*	*250*	***737***

### Analyses of data

#### Frequency of NF-Ds; relation to item difficulty and discrimination

Statistical analyses were performed using SPSS, version 15. We first calculated the frequency of distractors, which were selected by <1% (or <5%) of the candidates. While others set the limit at 5%, we also included analyses based on a more conservative definition of a distractor as non-functional when <1% of the candidates chose it. We additionally analysed the relation between the number of NF-Ds and the percentage of correct answers (P-value) as well as item discrimination.

#### Attractiveness of functional and non-functional distractors for low-achievers

As the next step, we determined to what degree distractors selected by <1% (or <5%) fulfilled their role to specifically attract low-performing candidates: We calculated the departure of the median values of candidates selecting such NF-Ds from the median value of the total group (i.e. delta-medians) and compared them with the respective delta-medians resulting from the choice of functional distractors (F-D). We refrained from calculating discrimination indices for single distractors as the sample of candidates selecting these distractors is mostly small and the scores are probably not normally distributed.

#### Calculative consequences of a reduction from 4 to 3 or 2 distractors

To analyse possible consequences of a reduction to 3 (or 2) distractors, respectively, several models were calculated on the basis of the 5 examinations conducted in 2005:

i) First, those distractors that were chosen the least and second least were identified.

ii) These distractors were then removed. Candidates who chose them were allocated to the remaining options using two extreme assumptions about their hypothetical behaviour:

a) Assuming that these candidates lacked knowledge and originally chose this distractor by chance, they were then allocated to one of the remaining options by chance [using the SPSS syntax: COMPUTE A1n = trunc(rv.uniform (0,5))+1).]

b) Assuming the other extreme, i.e. that these candidates chose this distractor, while oscillating between this specific distractor and the correct answer, the candidates were then allocated to the correct answer [using the SPSS syntax: if (A1 = 3) A1n = 5.]

These two extreme assumptions were expected to cover the range of possible outcomes, which would result from presenting <4 distractors.

According to this scheme, all items were reduced to 4 options by discarding the most rarely selected distractor (models A and B in the results section). Items were then subsequently reduced to 3 options by removing the second least chosen distractor (models C and D in the results section). No further changes were made to a question if either a distractor or 2 distractors had not been chosen by anyone (0%). The mean P-values (percentage of correct answers), selectivities (r) and reliabilities (Cronbach's alpha) of the original and the corresponding modified versions were then calculated. For r-values Fisher Z-transformations were performed prior to averaging, but no further transformations (e.g. logit transformation of P-values) were used, as these have been reported to not substantively alter results [6]. As we were mainly interested in the description of possible changes and their practical relevance, we also refrained from performing further statistical analyses.

#### Conduction and analysis of the expert survey

All 55 positively formulated best answer items (type A+) from the 'internal medicine/pharmacotherapy' examination in 2005 were presented to a sample of 37 experienced clinicians based at two university-based clinics of internal medicine. As MCQs authors are mostly recruited from university-based clinics, this clinician sample represented a typical target group of item writers. As MCQs from the Swiss Federal examinations are highly confidential because they are partly re-used for future examinations, we had to restrict the survey to 1 specialty and a limited sample of experts only.

The clinicians were provided with the following instruction: "Please mark the option, which according to your point of view is the most obviously for candidates to detect as 'wrong', with the number "1", and the option, which is the next obviously 'wrong' response with the number "2". If you think that the two options are equally unattractive, mark both options with a "1". In any case, please mark two options. The correct answer is labelled in order to facilitate your review." To limit the burden for experts, the 55 items were randomly split into 2 halves and each participant received either block A (27 questions) or block B (28 questions). Using this approach, each item was rated by at least 17 experts.

Based on items with at least 1 NF-D, a hit was defined as an expert's rating of one of the distractors as least plausible, which indeed had been selected by <1% (or 5%) of the candidates. If an expert chose two options as equally implausible, one of these was randomly picked out for this initial analysis. Associations between the proportion of the experts' hits and the number of NF-Ds were analysed by Pearson correlation. The mode of the experts' ratings was used as an approximation of an expert panel´s rating and the analyses were repeated.

The consequences of a reduction to 3 or 2 distractors based on the experts' voting were analysed. Again, the two extreme models described above were used. Mean P-values, r-values and Cronbach's alpha were calculated again, based on the 4-option or 3-option examinations resulting from these models.

## Results

### The frequency of non-functional distractors and relation to item difficulty and discrimination

Of 737 positively formulated best answer items from the examinations spanning 2005-2007, 30.3% (223 items) had 4 functional distractors (F-D), which were selected by ≥1% of the candidates. Correspondingly, 31.9% of the items had one, 23.1% had two, 11.3% had three, and 3.5% had four NF-D selected by <1% of candidates. From to the total number of 2948 distractors, 929 (31.5%) were selected by <1% of the candidates.

Following the definition of a distractor as non-functional if <5% of the candidates chose it, only 2.8% of the items (n = 21/737) had 4 F-D. Correspondingly, 10.2% of the items had one, 24.0% had two, 36.8% had three, and 26.2% had four NF-D selected by <5% of candidates. About two-thirds of the total number of distractors were selected by <5% of the candidates (n = 2014/2948, 68.3%).

**Figure 1:**
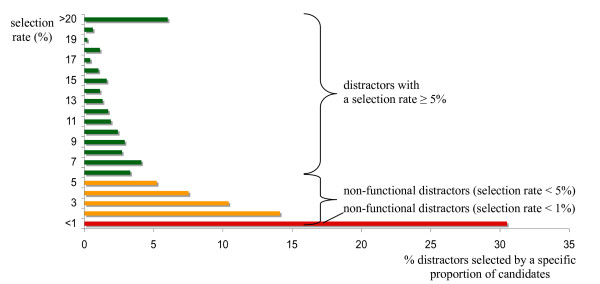
**Percentages of distractors with different selection rates (i.e. selected by specific proportions of candidates)**.

Among the distractors, 196 (6.6%) were never selected. Figure 1 depicts the percentages of distractors selected by specific proportions of candidates.

The easier an item (more candidates select the correct answer), the more distractors will be non-functional (and vice-versa). Items with 4 F-Ds selected by ≥1% from the examinations over the period 2005-2007 had a mean P-value of 67.5 and a mean discrimination of r = 0.20. These values were P = 78.1 and r = 0.19 for items with 1 NF-D, P = 82.4 and r = 0.18 with 2 NF-Ds, P = 93.6 and r = 0.15 for 3 NF-Ds, and P = 98.7 and r = 0.12 for 4 NF-Ds selected by <1%.

### Attractiveness of functional vs. non-functional distractors for low-achievers

**Figure 2:**
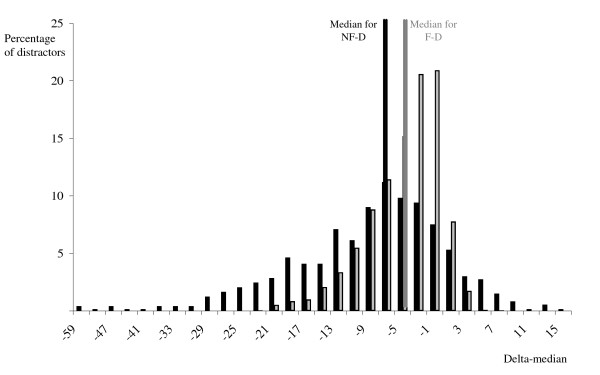
**The delta-median of the functional (grey bars) versus non-functional (black bars) distractors (F-D and NF-D)**.

Figure 2 depicts the delta-medians of F-Ds as well as NF-Ds selected by <1%. The medians of candidates who chose one of the F-Ds departed in the median by -3.9 points from the total candidate group median (range: -22.6 to 6.5). The medians of candidates who selected one of the NF-Ds departed in the median -7.6 points (range: -58.9 to 14.9). Comparable differences in medians were observed for NF-D selected by <5% (median: -6.5, range -58.9 to 14.9) compared to candidates who chose one of the FD (median: -2.3, range -16.7 to 3.9).

### Change in difficulty, discrimination and reliability: statistical approach (table 2)

#### Difficulty

With respect to all 5 examinations, a reduction to 3 distractors slightly increased mean percentage of correct answers (P-value), both when the candidates were randomly allocated to one of the other options or purposively allocated to the correct answer. This effect is more pronounced for a reduction to 2 distractors, especially following the assumption that candidates had oscillated between the specific distractor and the correct answer, and would consequently choose the correct answer should the specific distractor be missing.

#### Discrimination and reliability

In contrast, mean discrimination was almost unaffected, irrespective of the model underlying the reduction from 4 to 3 distractors (models A and B). Even a reduction from 4 to 2 distractors and allocating candidates to the correct answer resulted in only limited changes in discrimination. Similarly, reliability standardised for 100 items was little affected by a reduction to 3 distractors. A marked difference in reliability was only observed when keeping only 2 distractors and allocating the candidates to the right answer (table 2).

**Table 2:** Parameter changes related to different numbers of distractors (statistical approach)

Specialty	Model	% correct (P-value)*	Discrimination*	Reliability*	Standard Reliability**
Internal Medicine, Pharmacology (55 items)	*4 distractors*	*78.93*	*0.19*	*0.74*	*0.84*
	
	A) 3 distractors, random	79.20	0.19	0.73	0.83
	
	B) 3 distractors, right answer	80.11	0.18	0.72	0.82
	
	C) 2 distractors, random	80.20	0.18	0.71	0.82
	
	D) 2 distractors, right answer	82.76	0.14	0.62	0.75

Surgery (54 items)	*4 distractors*	*77.74*	*0.13*	*0.59*	*0.73*
	
	A) 3 distractors, random	77.91	0.13	0.59	0.73
	
	B) 3 distractors, right answer	78.44	0.13	0.58	0.72
	
	C) 2 distractors, random	78.78	0.13	0.58	0.72
	
	D) 2 distractors, right answer	80.75	0.11	0.54	0.68

Social and preventive medicine (41 items)	*4 distractors*	*76.32*	*0.11*	*0.50*	*0.71*
	
	A) 3 distractors, random	76.70	0.11	0.50	0.70
	
	B) 3 distractors, right answer	77.62	0.10	0.46	0.68
	
	C) 2 distractors, random	77.61	0.10	0.45	0.67
	
	D) 2 distractors, right answer	80.20	0.07	0.36	0.58

Pediatrics and gynaecology (43 items)	*4 distractors*	*75.73*	*0.14*	*0.57*	*0.76*
	
	A) 3 distractors, random	76.05	0.13	0.56	0.74
	
	B) 3 distractors, right answer	76.99	0.12	0.52	0.72
	
	C) 2 distractors, random	76.97	0.12	0.54	0.73
	
	D) 2 distractors, right answer	79.38	0.10	0.47	0.67

Dermatology, ophthalmology, otorhinolaryngology (57 items)	*4 distractors*	*79.69*	*0.22*	*0.77*	*0.86*
	
	A) 3 distractors, random	79.92	0.21	0.76	0.85
	
	B) 3 distractors, right answer	80.64	0.20	0.75	0.84
	
	C) 2 distractors, random	80.79	0.20	0.74	0.83
	
	D) 2 distractors, right answer	82.91	0.16	0.68	0.78

* These indicators related to the sample of positive A-type questions only, thus they differ from the respective indicators relating to the complete examination including negative best answer and extended matching questions.

** Reliability standardized for 100 items

### Expert survey

#### Proportion of hits

A hit was defined as an expert's identification of one of the NF-D as least plausible, so the following analyses were based on items with at least 1 NF-D (n = 34/55 items had at least 1 NF-D selected by <1%; n = 54/55 had at least 1 NF-D selected by <5%). Naturally, the chance of an expert 'correctly' marking a NF-D as implausible increases with the number of NF-Ds per item: Pearson correlation between the proportion of hits and the number of NF-Ds per item was r = 0.76 for those NF-Ds selected by <1% of the candidates (r = 0.82 for NF-Ds selected by <5%). Correspondingly, the range of hits varied between 10% and 100% for the correct detection of NF-Ds selected by <1% of the candidates with a mean of 64% (for NF-Ds selected by <5%, range = 20% - 100%, mean = 82%).

Regarding the 25 items with 1 or 2 NF-Ds only, 52% (range: 10 - 88%) of the experts on average correctly marked a NF-D selected by <1% of the candidates (for the 22 items with only 1 or 2 distractors selected by <5% of candidates, 61% of the experts correctly marked one of the NF-D, range: 20 - 88%).

Focusing on the recommendation of the *majority *of experts (i.e. mode of the 'expert panel'), a higher proportion of hits would result. The 'expert panel' correctly rated one the NF-D selected by <1% of the candidates as least plausible for n = 25/34 items (74% hits; n = 50/54 or 93% hits for NF-Ds selected by <5%).

For the 25 items with only 1 or 2 NF-Ds selected by <1% of the candidates, the 'expert panel' correctly marked one of the NF-D for 16 items (= 64% hits; n = 18/22 or 82% hits for items with 1 or 2 distractors selected by <5%).

### Change in difficulty, discrimination and reliability: expert recommendation (table 3)

#### Difficulty

Following the expert panels' recommendation of which distractor to discard, mean percentage of correct answers (P-values) would generally increase slightly more compared to the statistical approach, making the exam easier.

#### Discrimination and reliability

Changes in discrimination and reliability, which would result from models A, B and C according to the experts' recommendation (see table 3), also did not differ markedly from changes that would result from the pure statistically based approach (see table 2). Model D would lead to the most pronounced decrease of reliability and discrimination, and exceed that following the statistical approach.

**Table 3:** Parameter changes related to different numbers of distractors (expert recommendation)

Specialty	Model	% correct (P-value)*	Discrimination*	Reliability*	Standard. Reliability**
Internal Medicine, Pharmacology (55 items)*	*4 distractors*	*78.93*	*0.19*	*0.74*	*0.84*
	
	A) 3 distractors, random	79.49	0.19	0.73	0.83
	
	B) 3 distractors, right answer	81.21	0.16	0.66	0.78
	
	C) 2 distractors, random	81.08	0.17	0.69	0.80
	
	D) 2 distractors, right answer	85.07	0.11	0.53	0.67

* These indicators related to the sample of positive A-type questions only, thus they differ from the respective indicators relating to the complete examination including negative best answer and extended matching questions.

** Reliability standardised for 100 items

## Discussion

This study has integrated theoretical considerations regarding non-functional distractors (NF-D) and candidates' hypothetical behaviour in the absence of NF-D, in terms of the psychometric characteristics, as well as an expert survey regarding the detection of NF-D in high stakes medical examinations.

### The frequency of non-functional distractors and their attractiveness

In our study, only about a third of the items had 5 options that were selected by ≥1% of the candidates. A minority of about 3% of items had 5 options selected by ≥5% of the candidates. We believe that a distractor should instead be more conservatively defined as non-functional if <1% of the candidates select it, because in high stakes examinations for a selected sample of well-prepared candidates, distractors cannot generally be expected to attract more than 1% of the candidates. From our point of view, distractors attracting up to 5% of the candidates still serve their purpose as usually no more than 5% of the candidates sitting the Federal MCQ examinations are insufficiently prepared and fail. From this point of view, the rule that '3 options represents a natural limit in most circumstances' would not be supported: About 62% of the items had more than 2 functional distractors, which were selected by ≥1%. However, the rule does hold if applied to NF-D selected by <5% as only about 13% of the items had more than 2 FD according to this definition.

Rarely selected distractors can, nevertheless, fulfil a role in specifically attracting low-performing candidates, as these depart more clearly from the median compared to candidates selecting a 'functional' distractor. Therefore when reducing the number of distractors, the ability to attract poorly performing candidates and item content (e.g. distractor similarity to the correct answer; [16]), should also be appreciated, as has been proposed by others [e.g. [6]].

### Psychometric changes following a reduction in the number of options

Two different extremes were assumed regarding the hypothetical behaviour of candidates who chose NF-Ds: In case these NF-Ds were eliminated, they could randomly choose another option - or purposively choose the correct answer, from which they had originally been distracted.

The results indicated that the percentage of correct answers (P-values) would generally increase - making the examinations easier, consistent with previous reports [e.g. [9]]. However, increasing the 'easiness' of examinations could be balanced by adapting the threshold for a pass for candidates. We generally apply Rasch equating to maintain the performance required for passing the examinations stable. Thus, an analogous increase of the pass-fail limit would compensate for a change in difficulty of an examination due to the omission of distractors; so overall pass-fail rates should be unaffected. Furthermore, a reduction from 4 to 3 or even 2 distractors - based on item statistics - does not seriously affect discrimination, irrespective of the underlying assumption regarding the candidates' behaviour. Reliability would only markedly decrease following a reduction to 2 distractors and directing candidates to the correct answer. However, the use of more items while reducing the number of options could theoretically compensate for such changes. But as no more than 4-5 seconds will be saved per discarded distractor, a reduction from 4 to 3 distractors would only allow for the inclusion of 3-4 additional items per hour [6].

The approach described above allowed the estimation of the extreme consequences of option elimination. Yet, most candidates rely on 'educated guessing' as opposed to random choice [8], so the first model would underestimate their correct answer rate. The second model obviously represents an upper limit for their chance of getting a point and therefore, an intermediate model would probably be most realistic. Additionally in our study those distractors selected most rarely have been removed, but they do not need to be 'non-functional' in all cases. A model removing NF-Ds only - resulting in varying numbers of distractors per item - should therefore lead to intermediate results compared to those reported.

### Expert survey

Similarly to the results obtained in other studies [6,13], our survey indicates that experts often, but not always, detect non-functioning distractors. A reduction in the number of options based on the recommendation of the majority of experts leads to changes in difficulty, discrimination and reliability, which can be judged acceptable. Why item authors do not more precisely identify the options selected most rarely by candidates may partly be explained by the fact that the authors use expert knowledge for their appraisal whereas candidates are not yet at this expert stage and also use their "test wiseness", hidden cues and other item flaws to identify wrong options [8].

However, to restrict the number of options requires 'that it is the non-functioning option that would not be written' [13]. We therefore support existing recommendations to include all plausible options that occur to the authors as a first step [1], and the most appropriate distractors are then selected by a review committee in a second step [6]. For new items, this selection would merely be based on the items' content (plausibility), while distractors' selection rates can be taken into account in subsequent analyses. We also agree that not all items must have an identical number of distractors - as 'the key is the quality of distractors, not the number' [1]. Thus, when composing an examination, the review committee could also add alternative distractors themselves or accept items, which turn out to have only 3 (or even 2) plausible distractors, arguing that the 'natural limit' for plausible distractors of this item seems to have been reached. After the administration of an examination, psychometric analyses of the items would be performed as usual. As long as they showed sufficient discrimination, items with 1 or 2 NF-D could be re-used in future examinations by keeping just the F-D with sufficient selection rates. For items with insufficient discrimination, new distractors replacing the NF-D have to be generated before re-use - or the whole item has to be replaced.

*Limitations*: Parts of this study are based on theoretical, not experimental, analyses. In an experimental setting, the consequences of a reduction in the number of distractors on the performance of the candidates' sample as a whole (e.g. due to enhanced guessing effects) could be analysed more precisely. Nevertheless, our results correspond to those obtained from published experimental studies, which also report little influence on the psychometric item and test characteristics following a reduction in the number of options [e.g. [6,13,14]].

Analyses based on classical test theory share the limitation that they are dependent on the sample of items and participants [17]. With respect to our analysis, the item sample being analysed was quite large (> 700 items) covering a broad content domain (9 medical specialities), and the candidates represented the total group sitting the Swiss federal final examinations in 3 consecutive years. Due to reasons regarding confidentiality, the expert survey was however restricted to items from internal medicine and only involved a limited number of experts, so further analysis is required to determine whether these results are generalisable.

## Conclusions

This analysis supports and expands the results from previous studies by concentrating on high stakes medical examinations and simulating two extreme scenarios about the candidates' hypothetical behaviour following the elimination of rarely selected distractors. The results indicate that only a minority of about 3 - 30% of MCQs in the medical examinations under study had 4 functional distractors - depending on the threshold by which a distractor is defined as 'non-functional'. Only with a reduction to 2 distractors and assuming that candidates would switch to the correct answer in the absence of a rarely selected distractor, marked differences in reliability and difficulty (and to a lesser degree discrimination) are to be expected. Nevertheless, rarely selected distractors fulfil their role in attracting low-performing candidates.

As review committees seem to be more effective in detecting NF-Ds compared to single experts, item authors are still advised to write as many distractors as is feasible. The number of these distractors can then be reduced in a second step based on item statistics and/or an expert panel's recommendation to varying numbers of 3-4 or sometimes even 2 distractors, which are of high quality and sufficiently plausible.

## Competing interests

The authors declare that they have no competing interests.

## Authors' contributions

RK and RH designed the study. AR performed the data analysis and wrote the first draft of the paper, which was critically reviewed and revised by RK and RH. All authors read and approved the final manuscript.

## Authors' information

AR, RH and RK are psychologists, working as scientific co-workers at the Assessment and Evaluation Unit, Institute of Medical Education Bern, Switzerland.

## Pre-publication history

The pre-publication history for this paper can be accessed here:

http://www.biomedcentral.com/1472-6920/10/85/prepub
